# Ionic liquid-based electrolytes for CO_2_ electroreduction and CO_2_ electroorganic transformation

**DOI:** 10.1093/nsr/nwab022

**Published:** 2021-02-06

**Authors:** Xingxing Tan, Xiaofu Sun, Buxing Han

**Affiliations:** Beijing National Laboratory for Molecular Sciences, CAS Key Laboratory of Colloid and Interface and Thermodynamics, CAS Research/Education Center for Excellence in Molecular Sciences, Institute of Chemistry, Chinese Academy of Sciences, Beijing 100190, China; School of Chemistry and Chemical Engineering, University of Chinese Academy of Sciences, Beijing 100049, China; Beijing National Laboratory for Molecular Sciences, CAS Key Laboratory of Colloid and Interface and Thermodynamics, CAS Research/Education Center for Excellence in Molecular Sciences, Institute of Chemistry, Chinese Academy of Sciences, Beijing 100190, China; School of Chemistry and Chemical Engineering, University of Chinese Academy of Sciences, Beijing 100049, China; Beijing National Laboratory for Molecular Sciences, CAS Key Laboratory of Colloid and Interface and Thermodynamics, CAS Research/Education Center for Excellence in Molecular Sciences, Institute of Chemistry, Chinese Academy of Sciences, Beijing 100190, China; School of Chemistry and Chemical Engineering, University of Chinese Academy of Sciences, Beijing 100049, China; Shanghai Key Laboratory of Green Chemistry and Chemical Processes, School of Chemistry and Molecular Engineering, East China Normal University, Shanghai 200062, China

**Keywords:** ionic liquid, carbon dioxide, electrocatalysis, green synthesis, value-added fuels and chemicals

## Abstract

CO_2_ is an abundant and renewable C1 feedstock. Electrochemical transformation of CO_2_ can integrate CO_2_ fixation with renewable electricity storage, providing an avenue to close the anthropogenic carbon cycle. As a new type of green and chemically tailorable solvent, ionic liquids (ILs) have been proposed as highly promising alternatives for conventional electrolytes in electrochemical CO_2_ conversion. This review summarizes major advances in the electrochemical transformation of CO_2_ into value-added carbonic fuels and chemicals in IL-based media in the past several years. Both the direct CO_2_ electroreduction (CO_2_ER) and CO_2_-involved electroorganic transformation (CO_2_EOT) are discussed, focusing on the effect of electrocatalysts, IL components, reactor configurations and operating conditions on catalytic activity, selectivity and reusability. The reasons for the enhanced CO_2_ conversion performance by ILs are also discussed, providing guidance for the rational design of novel IL-based electrochemical processes for CO_2_ conversion. Finally, the critical challenges remaining in this research area and promising directions for future research are proposed.

## Teaser

The major advances in the electrochemical transformation of CO_2_ into value-added carbonic fuels and chemicals in IL-based electrolytes through the construction of C−H, C−C, C−O and C−N bonds were highlighted in this review.

## INTRODUCTION

Human society relies mainly on fossil fuels to meet the main energy demand since the industrial revolution. The use of fossil fuels as energy carriers and raw materials promotes the rapid development of society. However, the excessive exploitation of fossil fuels has given rise to the energy crisis and undesirable environmental changes [1,2]. The unrestrained combustion of these non-renewable fossil fuels also leads to a continuous increase of CO_2_ concentration in the atmosphere, which is >400 ppm today and is estimated to triple by 2040 [3]. The excessive emission of CO_2_ results in a series of environmental issues, such as global warming, rising sea levels and more extreme weather events. As a consequence, the utilization of abundant renewable energy is an urgent need and challenge for our society.

**Scheme 1.**
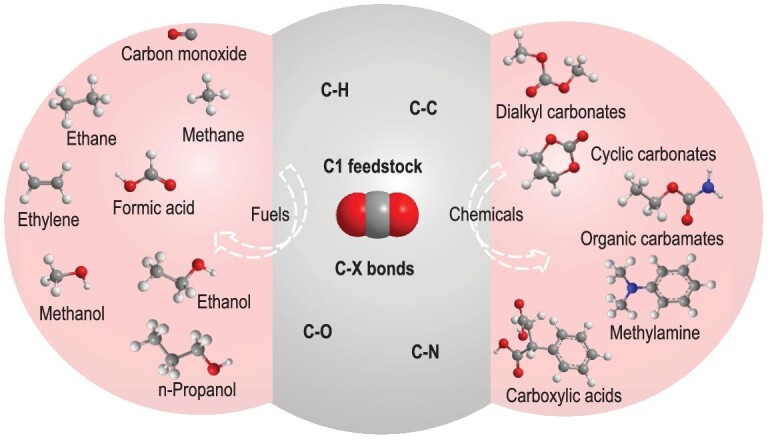
Value-added fuels and chemicals produced from CO_2_ transformation through the construction of various C−X bonds.

CO_2_ is not only one of the main greenhouse gases but also an abundant, non-toxic, non-flammable and renewable C1 resource. Producing fuels or chemicals using CO_2_ is an attractive way to achieve a carbon-neutral energy cycle [4]. As illustrated in Scheme 1, CO_2_ can be used as a feedstock to synthesize fuels and chemicals through the formation of various chemical bonds, such as C−H, C−C, C−O and C−N bonds [5,6]. CO_2_ reduction represents an essential approach for CO_2_ utilization, in which CO_2_ could be transformed into many platform chemicals through the construction of C−H bonds, such as hydrocarbons, acids and alcohols [7–9]. In addition, using CO_2_ as one of the reactants to synthesize valuable products is also an emerging strategy for CO_2_ conversion. When CO_2_ is used in carboxylation reactions, C−C bonds can be formed to produce valuable products, like carboxylic acids and organic carbonates [10,11]. The C−O or C−N bonds are established in CO_2_ cycloaddition reactions with different substrates (e.g. epoxides, aziridines or propargylic amines) to synthesize cyclic carbonates and oxazolidinone derivatives [12,13].

Although the exploitation of CO_2_ is particularly promising, the high thermodynamic stability and chemical inertness of CO_2_ make it difficult to activate, posing a huge challenge for CO_2_ conversion technology. In the past decades, a host of available pathways have been used to convert CO_2_ into various chemicals, including thermochemical, electrochemical, photochemical and biochemical pathways [9,10,12,14]. Among these methods, electrochemical conversion of CO_2_ has attracted tremendous attention recently, including direct CO_2_ electroreduction (CO_2_ER) and CO_2_ electroorganic transformation (CO_2_EOT). The electrochemical conversion of CO_2_ has moderate efficiency, simple reaction units and great potential for real industrial application. Moreover, the reaction direction, rate and efficiency can be easily tuned by adjusting electrode materials, electrolytes and the applied potential [15]. Especially, it can be performed under ambient conditions via renewable electricity. Therefore, the process is regarded as a convenient way to convert captured CO_2_ into value-added products and make it possible to store electrical energy in chemical form. To date, the electrochemical conversion of CO_2_ has been achieved in homogeneous or heterogeneous reactions [16,17]. The homogeneous electrocatalysts, such as metal-organic complexes, can interact with CO_2_ molecules through their unique active centers to exhibit remarkable selectivity toward the conversion of CO_2_. Thus, this has prompted extensive research attention since the 1970s [16]. However, the homogeneous catalyst systems suffer from complicated synthesis processes, high cost of catalysts, difficulty in post-separation and recycling, and toxic effects, which are disadvantageous for industrial applications [18]. Compared with homogeneous catalysis, heterogeneous catalysis is characterized by an easy synthesis of catalysts, prominent electrocatalytic activity, easy separation and recycling, and low toxicity. Therefore, CO_2_ electrochemical conversion in heterogeneous catalyst systems has been developed rapidly in recent years [19,20].

Various aspects of the electrocatalytic system have been explored to promote the development of CO_2_ conversion technology, including electrocatalysts, electrolytes and electrochemical cells [21–23]. As an important component in the electrocatalysis process, the electrolyte interacts with the electrode surface, reactants and intermediates, which play a key role in charge transport [22,24]. The differences in CO_2_ solubility, conductivity and viscosity are believed to have significant effects on the catalytic activity for CO_2_ER and CO_2_EOT. Aqueous electrolyte is one of the most common electrolytes, but the low CO_2_ solubility (0.033 mol L^–1^ CO_2_ in water under 298 K, 1 atm) and unsatisfactory applicable potential range hinder its practical application [25]. The organic electrolyte exhibits higher CO_2_ solubility and enhanced applicable potential, but its drawbacks (e.g. poor conductivity, toxicity and environmental hazards) should be assessed critically [22].

**Figure 1.**
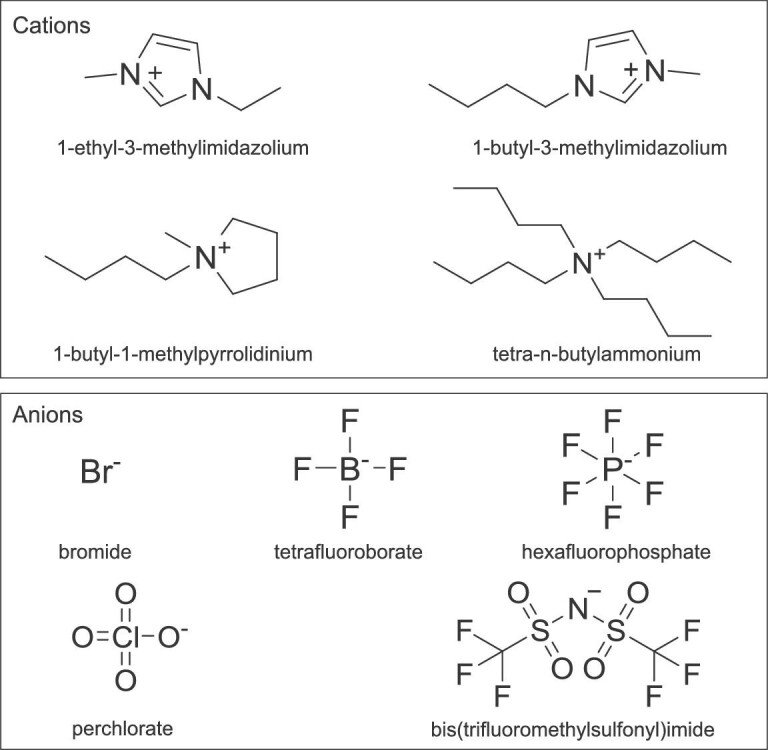
Some typical cations and anions of typical ILs for CO_2_ER and CO_2_EOT.

As a new type of green and chemically tailorable solvent, ionic liquids (ILs) have been proposed as highly promising alternatives for conventional solvents in many fields, such as material synthesis, gas adsorption and separation, electrochemistry, and catalysis [26,27]. ILs generally refer to low-melting salts consisting of an organic cation and an inorganic or organic anion [28]. The structures of some commonly used ILs are shown in Fig. 1. They have received tremendous interest due to having very low vapor pressure, high thermal stability, high ionic conductivity, high gas solubility and chemical diversity and tailorable ability [29]. In recent years, ILs have been studied extensively as electrolytes in many electrochemical reactions [30]. Many studies on CO_2_ capture also use ILs as CO_2_ absorbents because of their high CO_2_ solubility [26,31]. Therefore, ILs are considered an appealing alternative to aqueous and organic electrolytes in CO_2_ER and CO_2_EOT. The high absorption capacity of CO_2_, high intrinsic ionic conductivity and wide electrochemical potential windows of ILs are beneficial for CO_2_ conversion. It was reported that ILs could reduce the initial barrier of CO_2_ conversion through lowering the formation energy of CO_2_^•–^ intermediate. Moreover, the competing hydrogen evolution reaction (HER) could be suppressed in the presence of ILs, which might be favorable for improving the selectivity of CO_2_ conversion [25].

Over the past 10 years, we have witnessed heightened research activities and an increasingly deepened understanding of the electrochemical transformation of CO_2_ in IL-based electrolytes. Different high-quality review articles on the electrochemical transformation of CO_2_ are accessible, involving electrocatalysts, electrolytes and electrochemical devices. However, a timely and comprehensive review devoted to both the direct CO_2_ER and CO_2_EOT in IL-based electrolytes is lacking. Considering the rapidity of progress in this field, the recent advances in electrochemical transformation of CO_2_ in IL-based electrolytes catalyzed with heterogeneous catalyst systems are discussed in this review. The review will be carried out in the following three parts. The direct CO_2_ER into various platform chemicals in IL-based electrolytes will be presented in the first part. The second part will concentrate on the use of CO_2_ as a reactant to realize its electroorganic transformation into valuable products in IL-based electrolytes. We will discuss the involved ILs system, various types of applied electrocatalysts, electrochemical cells, products and reaction mechanisms. In addition, the challenges and perspectives for CO_2_ER and CO_2_EOT in the IL-based system will be outlined in the final section.

## CO_2_ER IN IL-BASED ELECTROLYTES

### Fundamentals of CO_2_ER

CO_2_ER is a proton-coupled multielectron transfer process, commonly involving 2, 4, 6, 8, 12 or even more electron reaction pathways [7,18]. Diversified reduction products with various carbon oxidation states can be obtained in the reduction process, including carbon monoxide (CO), formic acid/formate (HCOOH/HCOO^–^), methanol (CH_3_OH), formaldehyde (HCHO), methane (CH_4_), ethylene (C_2_H_4_), ethanol (CH_3_CH_2_OH), acetic acid/acetate (CH_3_COOH/CH_3_COO^–^) and n-propanol (C_3_H_7_OH). The electroreduction pathways for converting CO_2_ into the above products and the thermodynamic potential are displayed in Equations (1)–(6) (CO_2_ reduction potentials vs. standard hydrogen electrode (SHE) at pH 7) [7]. In terms of thermodynamics, it is readily accessible to reduce CO_2_ to these desirable products. Nevertheless, these reactions generally suffer from sluggish kinetics and low efficiency, which is related to the complicated reaction mechanism [32].

(1) \begin{eqnarray*} {\rm{C}}{{\rm{O}}_2} + 2{{\rm{H}}^ + } + 2{{\rm{e}}^-} &\to& {\rm{CO}} + {{\rm{H}}_2}{\rm{O}}\quad \nonumber\\ {E_0} &=& - 0.52\,{\rm{V}} \end{eqnarray*}

(2) \begin{eqnarray*} {\rm{C}}{{\rm{O}}_2} + 2{{\rm{H}}^ + } + {\rm{2}}{\rm e^-} &\to& {\rm{HCOOH}}\quad\nonumber\\ {E_0} &=& - 0.61\,{\rm{V}} \end{eqnarray*}

(3) \begin{eqnarray*} {\rm{C}}{{\rm{O}}_2} + 4{{\rm{H}}^ + } + 4{{\rm{e}}^-} &\to& {\rm{HCHO}} + {{\rm{H}}_2}{\rm{O}}\quad\nonumber\\ {E_0} &=& - 0.51\,{\rm{V}} \end{eqnarray*}

(4) \begin{eqnarray*} {\rm{C}}{{\rm{O}}_2} + 6{{\rm{H}}^ + } + 6{{\rm{e}}^-} &\to& {\rm{C}}{{\rm{H}}_3}{\rm{OH}} + {{\rm{H}}_2}{\rm{O}}\quad\nonumber\\ {E_0} &=& - 0.38\,{\rm{V}} \end{eqnarray*}

(5) \begin{eqnarray*} {\rm{C}}{{\rm{O}}_2} + 8{{\rm{H}}^ + } + 8{{\rm{e}}^-} &\to& {\rm{C}}{{\rm{H}}_4} + 2{{\rm{H}}_2}{\rm{O}}\quad\nonumber\\ {E_0} &=& - 0.24\,{\rm{V}} \end{eqnarray*}

(6) \begin{eqnarray*} 2{\rm{C}}{{\rm{O}}_2} + 12{{\rm{H}}^ + } + 12{{\rm{e}}^-} &\to& {{\rm{C}}_2}{{\rm{H}}_4} + 4{{\rm{H}}_2}{\rm{O}}\quad\nonumber\\ {E_0} &=& - 0.34\,{\rm{V}} \end{eqnarray*}

(7) \begin{eqnarray*} {\rm{C}}{{\rm{O}}_2} + {{\rm{e}}^-} \to {\rm{C}}{{\rm{O}}_2}^{ \bullet -}\quad {E_0} = - 1.90\,{\rm{V}} \end{eqnarray*}

(8) \begin{eqnarray*} 2{\rm{H}} + {}^{+ } {2}{{\rm{e}}^-} \to {{\rm{H}}_2}\quad {E_0} = - 0.42\,{\rm{V}} \end{eqnarray*}

According to the literature, the reaction of CO_2_ER can be conducted according to the following three steps [15]. The first step involves CO_2_ adsorption and activation, which is considered the most critical bottleneck in CO_2_ER. CO_2_ is a highly stable linear molecule with no electrical dipole, which makes CO_2_ adsorption on the catalyst surface difficult, and a large amount of energy is needed to activate CO_2_ [21]. As shown in Equation (7), transferring one electron to the CO_2_ molecule to form the key intermediate CO_2_^•–^ will initiate up to −1.90 V vs. SHE, which contributes to the high overpotential and an undesired major by-product H_2_ (Equation (8)). After the formation of CO_2_^•–^, the multiple proton-coupled electron transfers occur and generate diverse reduction products. However, the small difference in thermodynamic potential Equations (1)–(6) can result in low product selectivity. Finally, the products are desorbed from the catalyst surface [18].

The typical heterogeneous system for CO_2_ER consists of anode and cathode compartments separated by a proton exchange membrane [25]. Both the CO_2_ reduction reaction and HER take place at the cathode driven by electric energy over the catalyst. The oxygen evolution reaction (OER) occurs in the anode compartment. An efficient electrocatalyst can suppress HER to reduce the by-product of H_2_. The common heterogeneous electrocatalysts can be classified into four groups: metals/alloys, metal oxides and sulfides, metal-organic frameworks/complexes, and carbon-based materials. To better realize CO_2_ER, many studies have been devoted to developing heterogeneous electrocatalysts by means of surface engineering, chemical modification, doping and nanostructured strategy to improve the catalytic efficiency of CO_2_ER [15,33–35].

The electrolyte, especially in the cathode, also has an important influence on CO_2_ER. A CO_2_-containing electrolyte provides a source of CO_2_, enabling sufficient CO_2_ to be transported to the electrocatalyst surface. The electrolyte not only has close interactions with the electrocatalyst, adsorbed CO_2_ molecule and intermediates, but also undertakes the role of transporting charge species [22,36]. Therefore, the electrolyte with high solubility for CO_2_ and other reactants, appreciable electric conductivities and wide electrochemical potential widows, is conducive to CO_2_ conversion. The lower proton concentrations of electrolytes are beneficial in suppressing the competing HER and reducing the unwanted side-product H_2_ in the CO_2_ conversion process. Different kinds of electrolytes have been used for CO_2_ER, such as aqueous electrolyte, organic electrolyte and IL-based electrolyte [37]. Among them, IL-based electrolytes are particularly advantageous because they can lower the energy to form CO_2_^•–^ intermediate and show a suppression effect on the HER [38–41].

### CO_2_ER in IL-based electrolyte

In 2004, Zhao *et al.* reported the electrosynthesis of syngas by electrolyzing supercritical CO_2_ and water in 1-butyl-3-methylimidazolium hexafluorophosphate ([Bmim]PF_6_) electrolyte for the first time [42]. In addition to CO and H_2_, a small amount of HCOOH was also detected. In 2011, Rosen *et al.* found that in an electrocatalytic system with Ag cathode and 18 mol% 1-ethyl-3-methylimidazolium tetrafluoroborate ([Emim]BF_4_) solution electrolyte, the reduction of CO_2_ to CO could be conducted for at least 7 h with Faradaic efficiency (FE) of 96% and the overpotential was below 0.2 V [39]. They claimed that the IL electrolyte could reduce the energy of the CO_2_^•–^ intermediate probably by complexation, thus lowering the initial reduction barrier and contributing to the improved activity. This report was marked as an important breakthrough in the development of IL electrolytes for CO_2_ER, and since then the use of IL-based electrolyte in CO_2_ER has received extensive interest and much related research has been published.

Several properties of ILs including their structures, conductivity, viscosity, CO_2_ solubility, polarity and stability can influence catalytic performance. Therefore, the cations/anions, functional group or even the length of the alkyl chain of ILs should be considered when using them as the electrolyte. Up to now, the most commonly used class of ILs are imidazolium-based ILs [19,40,41,43].

#### Applied electrocatalysts

So far, a diversity of electrocatalysts has been developed for CO_2_ER in ILs. Metals are the most widely studied working electrode materials, including noble metals, transition metals and post-transition metals [15,21,44,45]. The noble metals Au, Ag and Pd are the representative model catalysts for CO_2_ER to produce CO. Zhu *et al.* reported the improved activity of Au nanoparticles in CO_2_ER by using [Bmim]PF_6_ as a more efficient COOH^*^ stabilizer [46]. Pt as the working electrode for CO_2_ER was also studied in different IL-based electrolytes [47,48]. Martindale and Compton reported that CO_2_ could be reduced into HCOOH in ILs bis(trifluoromethane)-sulfonimide (HNTf_2_) and 1-ethyl-3-methylimidazolium bis(trifluoromethylsulfonyl)imide ([Emim]NTf_2_) by using the pre-anodized Pt wire [48]. Considering the high cost and limited reserves of noble metals, earth-abundant and inexpensive transition metals such as Co, Cu and Mo have been considered as potential electrocatalysts for CO_2_ER [18,49,50]. Huan *et al.* reported the first Cu-based material for reduction of CO_2_ into HCOOH with high selectivity in [Emim]BF_4_/H_2_O electrolyte [51]. They proposed that ILs played a role in activating CO_2_. The combination of nanostructured porous dendritic Cu-based electrocatalysts with [Emim]BF_4_/H_2_O electrolyte also contributed to the excellent activity and high selectivity for HCOOH, indicating the importance of the electrolyte. Post-transition metals have also been investigated, such as Bi, In and Sn [52–55]. For example, Rosenthal *et al.* demonstrated that CO_2_ could be selectively reduced to CO with a high FE of 95% by using a Bi-based electrocatalyst combined with imidazolium ILs, while HCOOH tended to be the product when Bi was combined with a bicarbonate aqueous electrolyte [56]. They claimed that the CO_2_^•–^ intermediate at the electrode surface could be stabilized by the interface between Bi^0^ and Bi^3+^ sites, and imidazolium ILs might have a crucial influence in this pathway.

**Figure 2.**
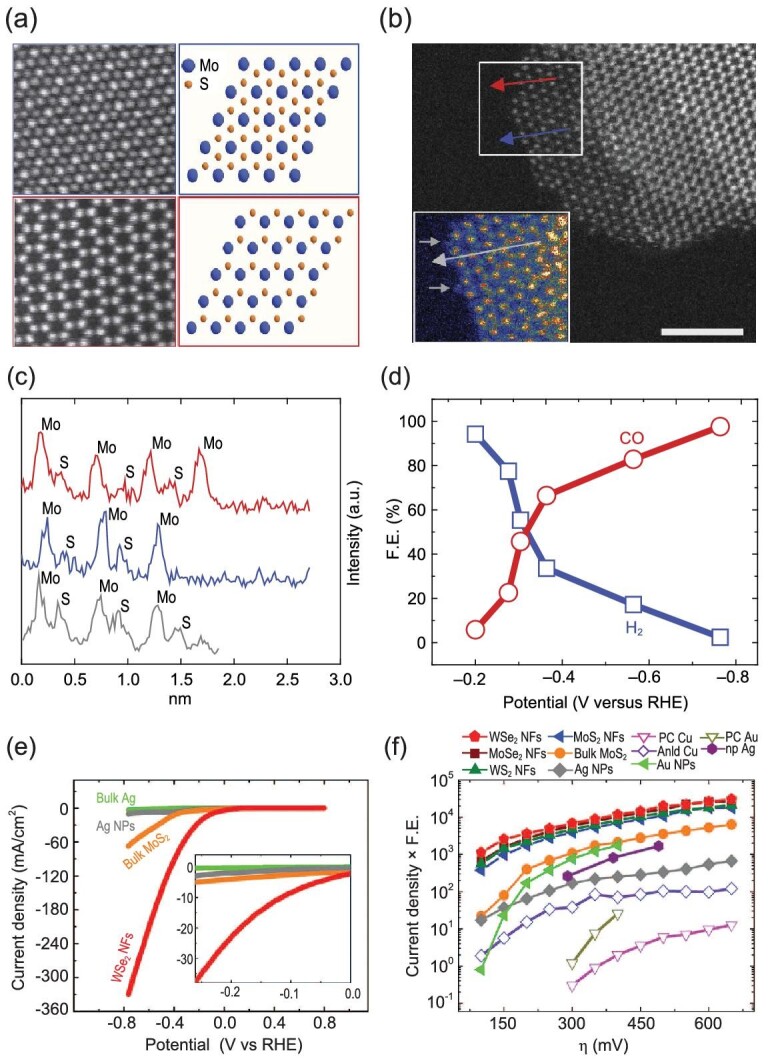
(a) The higher magnification high-angle annular dark-field (HAADF) images and the related schematic atomic models corresponding to the 1T (top) and 2H (bottom) type of MoS_2_. (b) Raw grayscale HAADF and false-color low-angle annular dark-field (LAADF) image (inset) of MoS_2_ edges. Scale bar, 5 nm. (c) The line scans of MoS_2_ flakes. (d) The FE of CO and H_2_ at different applied potentials. Adapted with permission from [61]. (e) Cyclic voltammetry (CV) curves for different catalysts in a CO_2_ environment. (f) Performance of different catalysts at different overpotentials (η). Adapted with permission from [38].

In addition, alloys, metal oxides and metal dichalcogenides are also promising electrocatalysts in IL-based electrolytes [43,57–59]. Sacci *et al.* reported that CO_2_ could be reduced to CO at −1.65 V vs. standard calomel electrode (SCE) by using Cu-Sn thin-film alloys in an imidazolium-based IL electrolyte [60]. They proposed that the synergistic interactions of the Cu-Sn cathode and the imidazolium cation contributed to the low overpotential. By using bulk molybdenum disulphide (MoS_2_) as the working electrode, CO_2_ was converted into CO with high current density and low overpotential (54 mV) in [Emim]BF_4_/H_2_O electrolyte, and the tunable mixture of H_2_ and CO (syngas) could be obtained by tuning the applied potentials (Fig. 2a–c) [61]. Moreover, this bulk MoS_2_ catalyst showed significantly higher catalytic performance for CO_2_ reduction than that of noble metals catalyst. Experimental and theoretical studies suggested that the Mo-terminated edges and the low work function of MoS_2_ contributed to the high catalytic activity for CO_2_ electroreduction. The two-dimensional (2D) nanoflake structures of different transition metal dichalcogenides (MoS_2_, WS_2_, MoSe_2_) were also explored for CO_2_ electroreduction in 50 vol% [Emim]BF_4_/H_2_O solution (Fig. 2d and e) [38]. WS_2_ nanoflakes showed superior CO_2_ electroreduction performance compared with the noble metal and other transition metal dichalcogenide catalysts with a high current density of 18.95 mA/cm^2^ at a low overpotential of 54 mV. The carbon-based materials are also promising heterogeneous catalysts in CO_2_ER. Sun *et al.* found that N-doped carbon (graphene-like) catalysts exhibited excellent activity and selectivity for CO_2_ER to CH_4_ by using [Bmim]BF_4_ as the electrolyte [62].

#### CO_2_ER in ILs

Imidazolium-based ILs are the most studied ILs for CO_2_ER due to their high CO_2_ capture ability [63]. Barrosse-Antle and Compton explored CO_2_ER in 1-butyl-3-methylimidazolium acetate ([Bmim]Ac), which exhibited a high CO_2_ solubility of 1520 mM [64]. The CO_2_ in [Bmim]Ac underwent a chemically irreversible, one-electron transfer to the radical anion CO_2_^•–^, and probably enabled the following formation of oxalate, CO and carbonate. CO_2_ could be reduced into HCOOH on pre-anodized Pt electrode in [Emim]NTf_2_ with HNTf_2_ as the proton source [48]. Kumar *et al.* found that metal-free carbon nanofibre (CNF) catalysts were quite efficient for CO_2_ER when [Bmim]BF_4_ was used as the electrolyte [65]. It exhibited a negligible overpotential (0.17 V) for electroreduction of CO_2_ to CO and much higher current density than that of Ag nanoparticles and bulk Ag film electrodes. Sun *et al.* reported that N-doped carbon (graphene-like) material/carbon paper electrodes could convert CO_2_ into CH_4_ in different IL electrolytes including [Bmim]BF_4_, [Bmim]PF_6_, 1-butyl-3-methylimidazolium trifluoromethanesulfonate ([Bmim]TfO), [Bmim]NTF_2_ and 1-butyl-3-methylimidazolium dicyanamide ([Bmim]DCA) [62]. They found that the ILs containing fluorine showed higher total current densities than the ILs without fluorine probably due to the strong interactions between CO_2_ and fluorine. In addition to the typical ILs with ‘common’ anions, Snuffin *et al.* designed and synthesized a novel IL 1-ethyl-3-methyl-imidazolium trifluorochloroborate ([Emim]BF_3_Cl), which was used to dissolve and electrochemically reduce CO_2_ [47]. With a Henry's constant of 4.1 MPa at 1 atm, the CO_2_ solubility was close to that reported in other ILs. A relatively positive reduction electrode potential of −1.8 V was recorded, and the current density reached 5.7 mA cm^−2^. They proposed that the BF_3_ could form a Lewis acid-base adduct BF_3_-CO_2_ with CO_2_, which weakened the C=O bond and prompted the reduction of CO_2_.

#### CO_2_ER in IL-based binary/ternary media

Although there are many advantages in using ILs as electrolytes in CO_2_ER, the relatively high cost and viscosity of ILs hinder their practical application. The use of IL-based mixtures such as binary/ternary media of ILs with water and/or organic solvents may provide an efficient medium for CO_2_ER.

**Figure 3.**
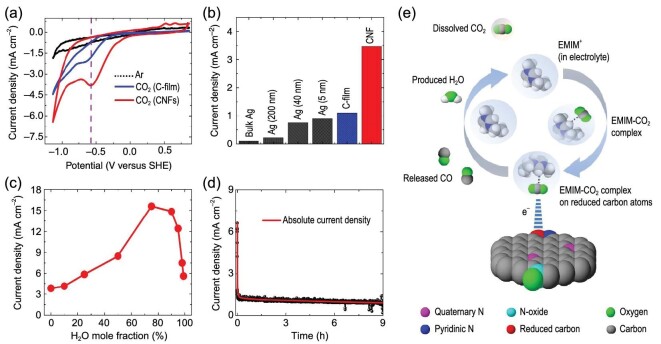
(a) CVs for CO_2_ reduction on carbon film electrode and CNFs electrode. (b) Absolute current density for CO_2_ reduction at different electrodes in pure [Emim]BF_4_ electrolyte. (c) Current density for CNFs catalyst with respect to H_2_O mole fraction (%) in [Emim]BF_4_. (d) Chronoamperogram for CNFs catalyst in pure [Emim]BF_4_. (e) Proposed schematic diagram for CO_2_ reduction mechanism. Adapted with permission from [65].

In 2012, Rosen *et al.* found that adding water into [Emim]BF_4_ (relatively hydrophilic) led to an increase of CO FE over Ag nanoparticle cathode [66]. The CO FE reached nearly 100% with 89.5 mol% water and 10.5 mol% [Emim]BF_4_, but the FE decreased at higher water concentrations probably due to the HER. In their following work, they studied the influence of water mole fraction in the electrolyte on CO_2_ER by using a metal-free CNF cathode (Fig. 3) [65]. Similar results were observed with an Ag nanoparticle cathode. Significantly, the current density for CO_2_ER to CO in 75 mol% water and 25 mol% [Emim]BF_4_ was about five times higher than that in pure [Emim]BF_4_ (Fig. 3b). They attributed these results to the decrease in pH and viscosity of [Emim]BF_4_ when mixed with water. With the addition of water, the hydrolysis of [Emim]BF_4_ led to a decrease in pH and a higher proton availability, thus accelerating the reduction of CO_2_. The decrease in viscosity after adding water also resulted in lower mass transport resistance. In addition, they proposed that the [Emim]^+^ cation could inhibit the HER caused by water addition. These indicate that the ratio of ILs in binary medium has a significant effect on CO_2_ER, and an optimum ratio is required to achieve an enhancement of CO_2_ reduction. In IL1-ethyl-3-methylimidazolium trifluoroacetate ([Emim]TFA) with 33% water binary medium, CO_2_ could be reduced to formate on In, Sn and Pb electrodes with high yield [52]. The peak charge on the voltammogram increased when 1 mL water was added into the ILs, but the peak charge decreased when the content of H_2_O increased up to 2 mL. Adding water into ILs leads to a dramatic decrease in solution viscosity [67], resulting in the accelerated diffusion of CO_2_ to the electrode surface and the improvement of CO_2_ER. However, at higher water content, the CO_2_ content is lower and the favorable effect of lower viscosity on CO_2_ reduction no longer prevails. Besides, the HER process seemed to be enhanced at higher water content.

**Figure 4.**
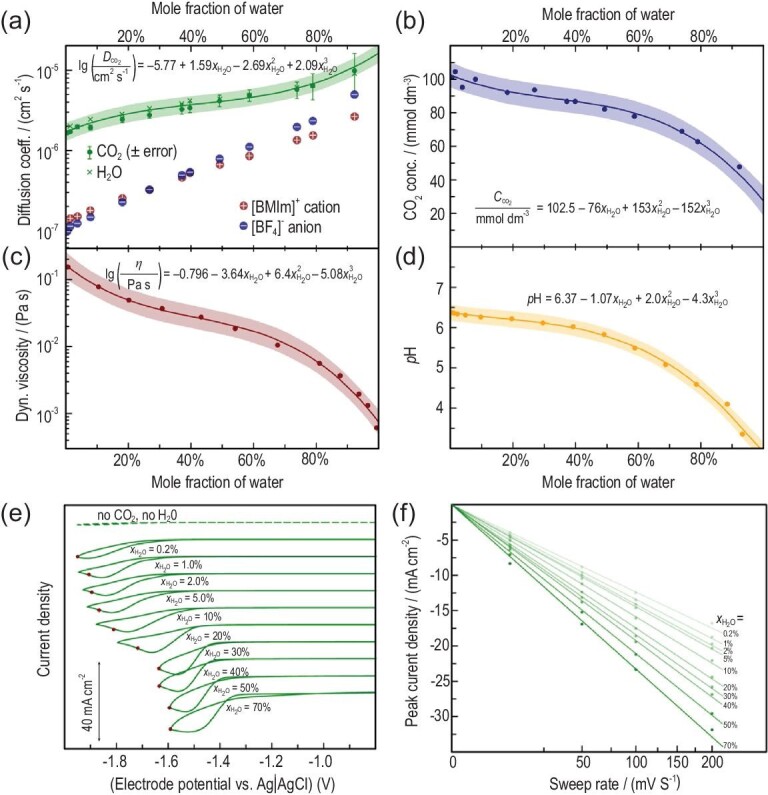
(a) Diffusion coefficients of CO_2_, H_2_O (overlapping), [Bmim]^+^ and [BF_4_]^–^ as determined by PGSE-NMR. (b) Dynamic viscosity of [Bmim][BF_4_]/water mixtures. (c) Solubility of CO_2_ in [Bmim][BF_4_]/water mixtures. (d) pH of [Bmim][BF_4_]/water mixtures with a different composition saturated with CO_2_. (e) CVs measured at a sweep rate of 50 mV s^–1^ with automatic IR-compensation. (f) The peak currents of CVs measured at different sweep rates. Adapted with permission from [68].

Rudnev *et al.* also conducted an in-depth study on the enhanced CO_2_ER in [Bmim]BF_4_/water binary medium by using several electrochemical methods combined with pulsed-gradient spin-echo (PGSE) nuclear magnetic resonance (NMR) spectroscopy (Fig. 4) [68]. They found that after the addition of water, the onset potential was lowered and the peak current increased. It was attributed to the increased availability of the protons and the enhanced diffusion of reacting species due to lower viscosity. FE 95.6 ± 6.8% was achieved for CO in a 50 mol% 1-ethyl-3-methylimidazolium trifluoromethanesulfonate ([Emim]TFO)/H_2_O electrolyte.

When ILs were dissolved in common organic solvents, the resulting IL/organic solvent binary medium may also exhibit some favorable properties for CO_2_ER, such as lower viscosity, high CO_2_ solubility, ionic conductivity and low price [69]. DiMeglio *et al.* found that adding [Emim]BF_4_ into the CO_2_-saturated MeCN solution resulted in an increased FE of CO formation (93 ± 7%) compared with that without ILs (48 ± 13%) [56]. Moreover, it led to an almost 40-fold increase in current density. When [Bmim]BF_4_ and [Bmim]PF_6_ were used to replace [Emim]BF_4_, the FE reached 95 ± 6% and 90 ± 9% in [Bmim]BF_4_/MeCN and [Bmim]PF_6_/MeCN, respectively. Further, the current density was higher than that in the case of [Emim]BF_4_/MeCN. They proposed that the proton source was most probably provided by deprotonation of the central imidazolium carbon of the cations of ILs. For example, the 1-butyl-2,3-dimethylimidazolium tetrafluoroborate ([Bmmim]BF_4_) with a methyl substituent at the imidazolium 2-position showed lower current density than [Emim]- and [Bmim]-based ILs, which was likely attributed to the difficulty of deprotonation of [Bmmim]BF_4_.

**Figure 5.**
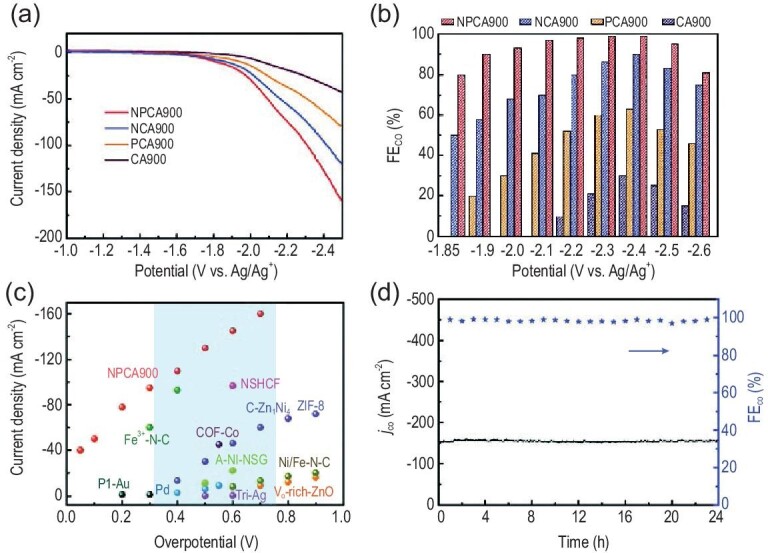
(a) Linear sweep voltammogram (LSV) curves over NPCA. (b) The FE(CO) for NPCA at different applied potentials. (c) The current density over NPCA compared with different catalysts. (d) Long-term stability of NPCA. Adapted with permission from [71].

Shi *et al.* reported CO_2_ER into CO in 1-butyl-3-methyl-imidazolium trifluoromethanesulfonates ([Bmim]CF_3_SO_3_)/propylene carbonate (PC) electrolyte with an Ag foil as a cathode [70]. Both [Bmim]CF_3_SO_3_ and PC exhibit high CO_2_ solubility. Commonly known, PC is a CO_2_ absorbent in industry and a common solvent used in organic electrochemistry. In this binary medium, Ag electrode showed a high FE of CO (90.1%) at −1.72 V (vs. Pt wire). Sun *et al.* found that Mo-Bi bimetallic chalcogenide electrocatalysts could efficiently catalyze the reduction of CO_2_ to CH_3_OH with a high FE of 71.2% and a current density of 12.1 mA cm^−2^ in 0.5 M [Bmim]BF_4_/MeCN [43]. The performance of CO_2_ electroreduction with Mo-Bi bimetallic chalcogenide/carbon paper electrode was also assessed in other common electrolytes/MeCN binary media, including [Bmim]PF_6_, 1-butyl-3-methylimidazolium perchlorate ([Bmim]ClO_4_), [Bmim]NTf_2_, tetra-n-butylammonium tetrafluoroborate (TBABF_4_), tetraethylammonium hexafluorophosphate (TEAPF_6_) and tetraethylammonium perchlorate (TEAClO_4_). In TBABF_4_, TEAPF_6_ and TEAClO_4_-based binary media, CO was the main product while the liquid product CH_3_OH was not detected. Chen *et al.* developed N, P-co-doped carbon aerogels (NPCA) catalysts for CO_2_ER (Fig. 5) [71]. The FE attained for producing CO reached up to 99.1% with a partial current density of 143.6 mA cm^−2^ by using 0.5 M [Bmim]PF_6_/MeCN as an electrolyte, which is much higher than the catalytic performance in 0.5 M KHCO_3_ aqueous solution (65.3% for FE and 45.5 mA cm^−2^ for current density). They attributed the significant performance advantage of [Bmim]PF_6_/MeCN electrolyte to the high CO_2_ solubility, lower reaction barrier via [Bmim-CO_2_]_(ad)_ complex formation, and the suppression of HER. The 0.1 mol L^–1^ super basic tetra alkyl phosphonium IL [P_66614_][124Triz] in MeCN has also been proved to be an effective medium for CO_2_ER [72].

**Figure 6.**
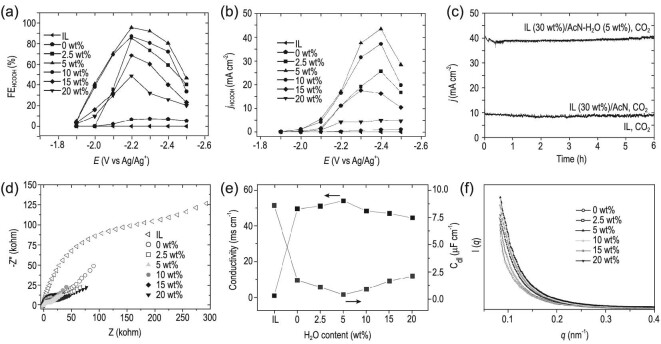
Effect of current density and FE using [Bmim]PF_6_ (30 wt%)/MeCN-H_2_O electrolytes with different H_2_O contents. (a) FE and (b) partial current density of HCOOH on Pb electrodes. (c) The dependence of current density over time on Pb electrodes. (d) Nyquist plots for the Pb electrode in various electrolytes. (e) Conductivities and double-layer capacitance, and (f) SAXS curves of [Bmim]PF_6_ (30 wt%)/MeCN-H_2_O mixtures. Adapted with permission from [53].

Zhu *et al.* investigated CO_2_ER in IL/MeCN/H_2_O ternary mixture electrolyte (Fig. 6) [53]. They found that the efficiency of CO_2_ER on Pb or Sn cathodes could be significantly enhanced by adding a small amount of H_2_O into [Bmim]PF_6_/MeCN or [Bmim]BF_4_/MeCN binary mixtures. By adjusting the composition of the ternary mixture, the performance and selectivity of CO_2_ER could be modulated (Fig. 6a–c). They also conducted CO_2_ER in ternary mixtures containing different IL components, including [Bmim]PF_6_, [Bmim]BF_4_, 1-butyl-3-methylimidazolium trifluoromethanesulfonate([Bmim]OTF), [Bmim]TFA, [Bmim]ClO_4_, [Bmim]DCA, 1-butyl-3-methylimidazolium thiocyanate ([Bmim]SCN), 1-butyl-3-methylimidazolium nitrate ([Bmim]NO_3_) and 1-butyl-3-methylimidazolium dihydrogen phosphate ([Bmim]H_2_PO_4_). Most of these IL/MeCN/H_2_O ternary media displayed excellent performance for HCOOH formation. Notably, when [Bmim]PF_6_ (30 wt%)/MeCN-H_2_O (5 wt%) was used as electrolyte, the FEs for HCOOH could reach 91.6% and 92.0% with a partial current density of 37.6 and 32.1 mA cm^−2^ on a Pb and Sn cathode, respectively. Combined with electrochemical methods and small-angel X-ray scattering (SAXS) (Fig. 6f), they illustrated that the presence of an appropriate amount of H_2_O in the ternary mixture resulted in higher solubility of CO_2_, increased conductivity, decreased double-layer capacitance and lowered onset potential, which contributed to the high current density. Yang *et al.* reported that CO_2_ could be converted into syngas on γ-In_2_Se_3_/carbon paper (CP) electrode in 30 wt% [Bmim]PF_6_/65 wt%MeCN/5 wt% H_2_O electrolyte [73]. They found that the composition of the electrolyte could not only have a significant effect on the total current density, but also affect the ratio of CO/H_2_ products. By adjusting the content of [Bmim]PF_6_ (5–70 wt%) and H_2_O (0–20 wt%), the CO/H_2_ ratio could be tuned from 9 : 16 to 24 : 1 and 2 : 3 to 24 : 1, respectively.

Some of the representative examples for CO_2_ electroreduction with different electrocatalysts in IL-based electrolytes have been summarized in Table 1. It can be seen that IL-based electrolyte affects the catalytic performance significantly.

**Table 1.** The representative examples of CO_2_ electroreduction with different electrocatalysts in IL-based electrolytes.

IL-based electrolytes	Catalysts	Reaction conditions	Main products (FE, %)	Current density (mA cm^−2^)	Ref.
[Emim]BF_4_/H_2_O (50 vol%/50 vol%)	WSe_2_ nanoflakes	−0.164 V (vs. RHE) overpotential 54 mV	CO (24)	18.95	[38]
18 mol% [Emim]BF_4_/H_2_O	Ag	−1.5 V (applied voltage), flow cell	CO (96)	−	[39]
10.7 g [Bmim]PF_6_ + 0.3 g H_2_O	Cu plank cathode	318.2 K and 8.95 MPa CO_2_, cell voltage<3.8 V^a^, high pressure undivided cell	Syngas CO (38.1) + H_2_ (50.6)	20	[42]
0.5 M [Bmim]BF_4_/MeCN	Mo-Bi bimetallic chalcogenide	−0.70 V (vs. RHE)	CH_3_OH (71.2)	12.1	[43]
[Emim]BF_3_Cl	Pt disk working electrode	−0.573 V (vs. SHE), negligible overpotential 0.17 V	CO (98)	−	[47]
[Emim]BF_4_/H_2_O (92/8% v/v)	Porous dendritic copper	−1.55 V (vs. Fc^+^/Fc)^b^	Formate (87)	6.5	[51]
[Bmim]PF_6_ (30 wt%)/MeCN^c^-H_2_O (5 wt%)	Pb	−2.30 V (vs. Ag/AgCl)	HCOOH (91.6)	37.6	[53]
[Bmim]PF_6_ (30 wt%)/MeCN-H_2_O (5 wt%)	Sn	−2.30 V (vs. Ag/AgCl)	HCOOH (92.0)	32.1	[53]
100 mM [Bmim][OTf]^e^ in MeCN	Bi nanoparticles	−2.0 V (vs. Ag/AgCl)	CO (96.1)	15.6	[54]
20 mM [Emim]BF_4_ in MeCN	Bi	−1.95 V (vs. SCE)^d^	CO (95)	5.51	[56]
4 mol%/96 mol% [Emim]BF_4_/H_2_O	MoSeS alloy monolayers	−1.15 V (vs. RHE)	CO (45.2)	43	[57]
4 mol%/96 mol% [Emim]BF_4_/H_2_O	MoS_2_	−0.764 V (vs. SHE)	CO (98)	65	[61]
[Bmim][CF_3_SO_3_]/PC^f^	Ag	−1.72 V (vs. Pt wire)	CO (90.1)	4.6	[70]
0.5 M [Bmim]PF_6_/MeCN	N,P-co-doped carbon aerogels	−2.4 V (vs. Ag/Ag^+^)	CO (99.1)	143.6	[71]
0.1 M [P_66614_][124Triz]^g^/MeCN	Ag	−0.7 V overpotential 0.17 V	0.05 mmol Formate (95)	–	[72]
30 wt%[Bmim]PF_6_/65 wt%	γ-In_2_Se_3_/CP^h^	−2.3 V	1:1 CO/H_2_ (90.1)	90.1	[73]
MeCN/5 wt%H_2_O		overpotential 220 mV	CO (96.5)	55.3	

^a^Cell voltage, ^b^ferrocene/ferrocenium, ^c^acetonitrile, ^d^standard calomel electrode, ^e^1-butyl-3-methylimidazolium trifluoromethanesulfonate, ^f^propylene carbonate, ^g^trihexyltetradecylphosphonium 1,2,4-triazolide, ^h^carbon.

### Mechanistic understanding of CO_2_ER in IL-based electrolyte

As shown above, many studies demonstrated that ILs were efficient media in CO_2_ER with excellent catalytic reactivity and selectivity (Table 1). Moreover, the designability of ILs permits tailoring the electrolyte to achieve optimal conditions for CO_2_ER. Therefore, a mechanistic understanding of IL-based electrolyte, especially the role of IL components, is important for the rational design of a new IL-based electrocatalytic system for CO_2_ transformation.

**Figure 7.**
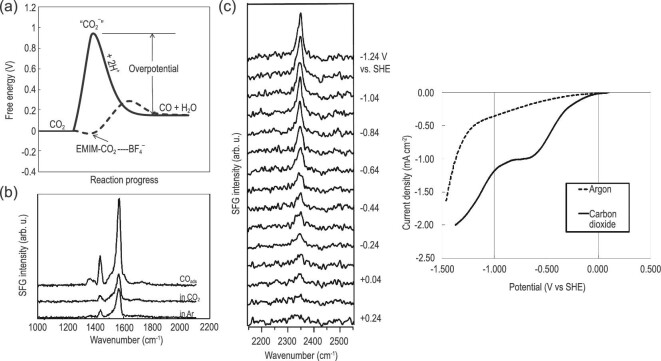
(a) A schematic of how the free energy of the system changes during the reaction CO_2_ + 2H^+^ + 2e^–^ ⇌ CO + H_2_O in water or acetonitrile (solid line) or [Emim]BF_4_ (dashed line). Adapted with permission from [39]. (b) SFG spectra of a platinum catalyst in [Emim]BF_4_. (c) Series of SFG spectra (left) and LSV (right) taken during CO_2_ electrolysis in [Emim]BF_4_ containing 90 mM water. Adapted with permission from [74].

In heterogeneous systems, CO_2_ER occurs at the surface of electrocatalysts. Therefore, the understanding of the electrocatalyst-IL interface is imperative. In 2011, Rosen *et al.* reported that a 96% selectivity to CO was achieved in an 18 mol% [Emim]BF_4_ solution with Ag cathode at a low overpotential, much higher than the ∼80% selectivity to CO in the absence of IL [39]. They proposed that IL could lower the energy of the (CO_2_)^–^ intermediate, probably by the formation of a complex between the IL and (CO_2_)^–^, resulting in a low-energy pathway for CO_2_ conversion (Fig. 7a). They further used sum-frequency generation (SFG), an effective technique for probing the solid–liquid interface, to explore the molecular structures at electrode interfaces to figure out the cause of this selectivity enhancement (Fig. 7b and c) [74]. Pt electrode was used for these *in situ* spectroscopic examinations since it is almost inactive for converting CO_2_ into CO, thus the enhancement in CO_2_ conversion could be readily detected. The results demonstrated that the formation of CO was observed in the presence of [Emim]BF_4_ and the formation of H_2_ was suppressed compared with aqueous systems. The SFG spectrum of Pt catalyst in [Emim]BF_4_ presented a CH_3_ bending mode at ∼1430 and a ring stretching mode at ∼1570 cm^−1^ (Fig. 7b), implying that a layer of [Emim]^+^ was located at the Pt electrode surface during electrolysis. The LSVs showed that no CO production was detected at a potential more negative than −0.8 V in the absence of [Emim]^+^, indicating that the adsorbed [Emim]^+^ could reduce the overpotential. New species with a peak at 2348 cm^−1^ appeared in LSV (Fig. 7c), which was presumed to be the formation of an [Emim-CO_2_]-BF_4_ complex. Then, the generation of CO was started at ∼−0.25 V vs. SHE, which was a low-energy pathway. Finally, they proposed that the adsorbed cation was used as a co-catalyst for converting CO_2_ to CO.

Quantum molecular dynamics simulations were used to reveal the role of ILs in observed high-CO_2_ reduction reaction. Asadi *et al.* reported that the complex [Emim-CO_2_]^+^ was most likely formed by the binding of CO_2_ with [Emim]^+^ via the C4/5 protons rather than through the C2 proton [61]. Moreover, the complex could be stabilized by H_2_ bonding. In the CO_2_ER, the complex [Emim-CO_2_]^+^ could be potentially physiosorbed at the negatively charged MoS_2_ cathode. This created a close encounter between CO_2_ molecules and MoS_2_ surface, resulting in an increment of local CO_2_ concentration near the cathode surface. Additionally, the existence of [Emim]^+^ was considered to reduce the reaction barrier for electrons transferring into CO_2_. Lim *et al.* indicated that the cations and anions in ILs could stabilize surface-bound intermediates to form a suitable microenvironment, thereby lowering the energy barrier and improving the CO_2_ reduction kinetics [45]. Thus, ILs were thought to play a crucial role in lowering the overpotential of CO_2_ reduction.

It was reported that imidazolium-based ILs can interact with CO_2_ by physical absorption, which can serve as both robust electrolytes and CO_2_ activation promoters [75]. The kinds of anions have a significant effect on the CO_2_ activation. The ILs containing fluorine such as [Bmim]BF_4_, [Bmim]PF_6_ and [Bmim]NTf_2_ exhibited much higher activity than the ILs without fluorine, which is partly because fluorine has strong interaction with CO_2_ [76]. Additionally, it leads to higher CO_2_ solubility in the electrolyte, which can avoid mass transport limitation in the electrolysis [77].

## CO_2_EOT WITH ORGANIC COMPOUNDS IN IL-BASED ELECTROLYTES

CO_2_ can also be utilized as a C1 synthon/building block to electrosynthesize valuable chemicals, which is another efficient pathway for CO_2_ utilization [5]. By the electrochemical reactions with different substrates, like epoxides, alcohols, amines, aryl halides and olefins, CO_2_ can be converted into various kinds of products, including cyclic carbonates, dialkyl carbonates, carbamates and carboxylic acids [78–81]. In particular, some reactions are thermodynamically unfavorable without external energy, and thus the efficient reaction routes conducted by thermal catalysis are very limited. Using an electrochemical method to synthesize organic molecules has various advantages, such as mild conditions, high functional group tolerance and innate scalability and sustainability [82]. The general pathway of the CO_2_EOT involves the generation of electro-induced radical/anion from CO_2_ and/or substrates, and then the radical/anion reacts with other substrates to yield various compounds. Several studies have further shown that ILs have a stabilization effect on the electro-induced CO_2_ molecule or substrates radical/anion. Combined with the high CO_2_ solubility and favorable electrochemical properties, ILs are considered as a green alternative reaction medium to volatile organic solvents for CO_2_EOT [13,83]. In this section, we will review the use of CO_2_ as a reactant in IL-based reaction media for electrosynthesis of value-added chemicals.

### Electrosynthesis of organic carbonates

Organic carbonates (especially cyclic carbonates and dialkyl carbonates) have attracted extensive attention owing to their wide usage as polar aprotic solvent, intermediate for polycarbonate and electrolyte in batteries [84,85]. Electrocatalytic fixation of CO_2_ to epoxides or alcohols to yield organic carbonates via C−O bond formation can avoid the use of toxic phosgene or CO, providing a green and atom economy pathway for the synthesis of organic carbonates.

Cyclic carbonate synthesis from CO_2_ and epoxides in pure ILs without additional supporting electrolyte and catalyst was reported by Deng and coworkers [78]. The reaction was performed in an undivided cell under mild conditions with a Cu working electrode and an Al or Mg rod sacrificed anode. The performance of cycloaddition of CO_2_ to different epoxide substrates (propylene oxide, epichlorohydrin and styrene oxide) was tested. The best performance was achieved by utilizing propylene oxide as substrate and [Bmim]BF_4_ as reaction media resulting in a 92% conversion and 100% selectivity to the desired product (cyclopropylene carbonate). CO_2_ underwent a one-electron reduction to generate the CO_2_^•–^ radical anion, and then reacted with the activated substrate to yield the corresponding cyclic carbonate. Wang *et al.* reported the electrosynthesis of cyclic carbonates from CO_2_ and diols in ILs in an undivided cell under mild conditions (1 atm, 50^o^C) [86]. When CO_2_ and 1,2-butanediol were used to synthesize butylene carbonate, the highest yield of 12% was achieved in [Bmim]BF_4_ with an Mg anode and a Cu cathode.

Zhang *et al.* found that the electrochemical activated CO_2_ in ILs could react with alcohol in the presence of an alkylating agent to generate dialkyl carbonates [79]. They found that CO_2_ was reduced to the anion radical CO_2_^•–^ in [Bmim]BF_4_ in an undivided cell. Especially, a more positive potential was recorded than that in organic solvents, which possibly attributed to the stabilization effect from CO_2_^•–^-[Bmim]^+^ ion-pairing. After adding CH_3_I as the alkylating agent, the dimethyl carbonate (DMC) was obtained by the reaction of CO_2_^•–^ with CH_3_OH. Cathodic material screenings revealed that Cu and Ag were more efficient than Ti, Ni and stainless steel with good yields of 73% and 74%, respectively. Different alcohol substrates were screened and the results showed that primary alcohol and secondary alcohol gave 33%–73% yields toward corresponding carbonates, while tertiary alcohol and phenol were unreactive. Wu *et al.* reported the electrosynthesis of dialkyl carbonates from CO_2_ and alcohols through electrogenerated N-heterocyclic carbenes [87]. With [Bmim]BF_4_ as the solvent and N-heterocyclic carbenes precursor, 90% conversion and 96% selectivity of benzyl methyl carbonate were achieved from CO_2_ and benzyl alcohol on Ti cathode. The other primary alcohols and secondary alcohols were also used as the substrates to react with CO_2_ to give the corresponding dialkyl carbonates. Moreover, various electrode materials were investigated to improve the yield of DMC. The porous nanostructure composite electrode consisting of Cu skeletons and platinum shells and Ag-coated nanoporous Cu composites electrode gave a slight improvement with yields of 81% and 80%, respectively [88,89]. A 76% yield was obtained using an In electrode [90].

To avoid the use of toxic alkylating agent CH_3_I, Yuan *et al.* proposed an IL-CH_3_OK-methanol system for the synthesis of DMC [91]. The electrochemical conversion of CO_2_ and CH_3_OH was conducted in IL electrolyte with Pt as electrodes and CH_3_OK as co-catalysts in an undivided four-neck bottle cell. They screened various ILs, including [Bmim]Br, [Emim]Br, [Bmim]Cl, [Bmim]OH, [Bmim]BF_4_ and [Emim]BF_4_. The highest yield of 3.9% for DMC with 88.4% selectivity was achieved in [Bmim]Br electrolyte. The anions of ILs were thought to have an important effect on the conversion. When ethanol was used as the substrate, diethyl carbonate was also synthesized in this electrochemical conversion process with a 0.4% yield. Instead of an undivided cell, a filter-press electrochemical cell with divided anodic and cathodic compartments was used in this IL-CH_3_OK-methanol system to better investigate the behavior of DMC electrosynthesis from CO_2_ [92]. Using a Nafion 117 membrane as the cationic exchange membrane, a 12.5% yield of DMC was obtained. A series of experiments were performed to study the influence of [Bmim]Br on the DMC electrosynthesis, and it revealed that [Bmim]Br might play a catalytic role in the process besides being used as an electrolyte. Nevertheless, further in-depth research is required to elucidate the reaction mechanism and ascertain possible specific roles of each components, especially CH_3_OK and IL. The influence of the membrane (anion, cation exchange membrane and without the use of membrane) in this IL-CH_3_OK-methanol system was investigated in the following work [93].

A [Bmim]Br-propylene oxide-methanol system was also developed to electrochemically convert CO_2_ into DMC, achieving yields of 75.5% and 37.8% on Pt electrode in the related research [94,95]. Though a high yield was obtained, this route is inconsistent with the green pathway of CO_2_ conversion due to the use of carcinogenic propylene oxide. To avoid the use of toxic additives and simplify the separation system, further work on the CO_2_EOT to DMC without any additives was also conducted. However, the yield of DMC was not satisfactory. Different ILs and cathodes were screened. The maximum yield of DMC achieved with Pt-graphite electrode in1-benzyl-3-methylimidazolium chloride([Bzmim]Cl)-methanol-CO_2_ system was only 3.8%. Considering the advantages of a basic medium in the absorption and activation of CO_2_, the amino-functionalized ILs were also developed to generate DMC [96]. Using 1-(3-aminopropyl)-3-methylimidazolium bromide as an electrolyte, a 2.5% yield with 94.5% selectivity of DMC was obtained with graphite electrode without adding any additives.

### Electrosynthesis of organic carbamates

Organic carbamates are important kinds of chemicals that have been extensively used as pharmaceuticals, agrochemicals and amine-protecting groups [97]. A phosgene-free process that uses CO_2_ as a C1 synthon to construct C−N bonds by electrochemical methods provides a green synthetic route for organic carbamates [98]. Feroci *et al.* reported the electrochemical fixation of CO_2_ with amines in ILs to synthesize organic carbamates [80]. Electrolysis of CO_2_-saturated [Bmim]BF_4_ solution containing amines was conducted in a divided glass cell at 55°C. Then, the aliphatic or aromatic amines could react with the cathodic activation of CO_2_ after adding EtI as an alkylating agent to yield corresponding carbamates. Cathodic material studies revealed that Pt cathodes were more efficient than Cu and Ni cathodes in the electrosynthesis of organic carbamates from CO_2_ and amines, with a maximum yield up to 80%. The authors proposed that the nucleophilicity of amines had a strong influence on the yield of carbamates. The primary and secondary aliphatic amines afforded carbamates with good yields of 73%–87%, while aniline showed a low yield of 38%.

### Electrocarboxylation

Electrocarboxylation of CO_2_ and organic compounds is an essential strategy for CO_2_ fixation via the construction of C−C bonds. Moreover, the use of CO_2_ as an alternative synthon to toxic and hazardous chemicals (e.g. phosgene and cyanides) provides a green route to synthesizing carboxylic acids and their derivatives. Different kinds of substrates, such as alkenes, alkynes, ketones and organic halides, undergo electrocarboxylation with CO_2_ to yield corresponding carboxylated products. Some studies focused on using ILs as a reaction media to reduce the use of volatile solvents and enhance efficiency.

Lu *et al.* developed the electrocarboxylation of activated olefins in CO_2_-saturated [Bmim]BF_4_ solution in an undivided cell under mild conditions [99]. Electrochemically reduced ethyl cinnamate reacted with CO_2_ to yield monocarboxylic acids as the main carboxylated product, as well as the by-product saturated esters. Screening on different cathodic materials (stainless steel, Ti, Cu, Ni) found that stainless steel was the most effective cathodic material, giving a 41% yield of monocarboxylic acid under optimized conditions (50°C, 1 atm CO_2_). This method was extended to other olefins, achieving the corresponding monocarboxylic acid with moderate yields of 35%–55%. Yuan *et al.* performed the electrochemical dicarboxylation of aryl-substituted alkenes and CO_2_ in an undivided cell with ILs as supporting electrolytes under room temperature [100]. Using styrene as a model molecule, the effects of various experimental parameters, including electrocatalysts, supporting electrolyte, CO_2_ pressure and concentration of substrates, were investigated to obtain optimal reaction conditions. Screening on electrode materials indicated a significant dependence of the activity on both cathode and anode materials, in the order Pt > Ni > Cu > Cu-Sn alloy and Al > Mg > Zn. The supporting electrolyte also showed an important influence on the electrocarboxylation, as the yield of 2-arylsuccinic acids decreased depending on both cation and anion, in the order [Bu_4_N]^+^ > [Et_4_N]^+^ and Br^−^ > Cl^−^ > I^−^. The electrolysis of styrene and CO_2_ in 0.05 mol L^−1^ n-Bu_4_NBr-DMF solution on Ni cathode and Al anode gave a principal product 2-phenylsuccinic acid in high yield and selectivity (87%, 98%), accompanied by by-product 3-phenylpropionic acid. This electrochemical route was extended to various aryl-substituted alkenes and gave the corresponding 2-arylsuccinic acids with yields of 50%–87%. They extended this method to electrocarboxylation of arylacetylenes [101]. The electrochemical dicarboxylation of phenylacetylene and its derivatives was conducted in a [Bu_4_N]Br-DMF electrolyte system in an undivided cell with Ni cathode and Al anode, and the corresponding aryl-maleic anhydrides and 2-arylsuccinicacids were generated with high total yields of 82%–94%.

**Figure 8.**
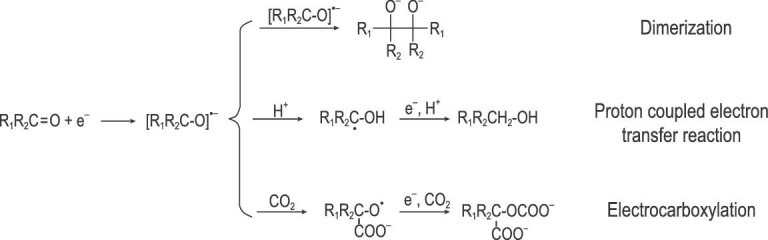
Competing reaction pathways for the electroreduction of aromatic ketones under a CO_2_ atmosphere. Adapted with permission from [104].

The electrocarboxylation of aromatic ketones with CO_2_ in [Bmim]BF_4_ in an undivided cell was reported by Feng and co-workers [102]. Various experimental parameters including temperature, electrode material, substrate concentration, current density, charge passed and working potential were screened to obtain the optimized conditions. The electrolysis of CO_2_-saturated [Bmim]BF_4_ solution containing a definite concentration of acetophenone or electron-donating substituted acetophenone was conducted with Pt cathode and Mg anode at 50°C, followed by adding the alkylating agent CH_3_I to afford the corresponding α-hydroxycarboxylic acid methyl ester with yields of 56%–62%. The corresponding alcohols were obtained as the main by-products. Zhao *et al.* studied the influence of proton availability in ILs on product distribution of electrocarboxylation of acetophenone with CO_2_ [103]. They revealed that dry 1-butyl-1-methylpyrrolidinium bis(trifluoromethylsulfonyl)imide ([BmPyrd]TFSI) with a low proton availability was an appropriate medium for this electrocarboxylation system to give 2-hydroxy-2-phenylpropionic acid with a good yield of 98%. The competing reactions are not conducive to the electrocarboxylation (Fig. 8) and some studies suggested that the product distribution depends strongly on the medium. In a following work, they further explored the influence of the nature of substrates and IL anions on the electrocarboxylation of aromatic ketones under CO_2_ atmosphere [104]. A highest yield of 40.7% (2-([1,10-biphenyl]-4yl)-2-hydroxy-2-phenylacetic acid) was achieved from electroreduction of 4-phenylbenzophenone in 1-butyl-2,3-dimethylimidazolium tris(pentafluoroethyl)trifluorophosphate ([Bmim]FAP).

Niu *et al.* reported the electrocarboxylation of benzyl chloride with CO_2_ in [Bmim]BF_4_ in an undivided cell [105]. The Ag cathode exhibited a higher yield of phenylacetic acid than the Cu or Ni cathode, while the sacrificial anode showed no obvious effect on the yields. They proposed that the benzyl chloride underwent a cathodic reduction to Ph_2_CH^−^, and then reacted with CO_2_ through a nucleophilic reaction. The difference between the reduction peak potential of CO_2_ and PhCH_2_Cl was influenced by the cathode materials. A closer reduction potential at the Cu or Ni electrode than the Ag electrode could bring an interference of CO_2_ reduction on the electrocarboxylation of PhCH_2_Cl, resulting in the poor yields of phenylacetic acid. The electrolysis of benzyl chloride in CO_2_-saturated [Bmim]BF_4_ with Ag cathode and Mg anode at 0.1 MPa CO_2_ and 50°C, followed by adding anhydrous K_2_CO_3_ and CH_3_I, afforded the phenylacetic acid with a yield of 45%. They also found that the residual water had an unfavorable impact on this electrocarboxylation system, leading to the generation of undesirable toluene product. Hiejima *et al.* tried to promote the electrocarboxylation of α-chloroethylbenezene in N,N-diethyl-N-methyl-N-(2-methoxyethyl)ammonium bis(tri-fluoromethanesulfonyl)amide (DEME-TFSA) IL with compressed CO_2_ [106]. The diffusion coefficient of α-chloroethylbenezene was improved at high temperature and pressure. The promotion in the substrate diffusion might contribute to the increase of current efficiency. Tateno *et al.* reported an IL/supercritical CO_2_ system for the electrocarboxylation of a variety of organohalides [107]. They explored the effect of CO_2_ pressure on electrocarboxylation and revealed that the efficiency was improved in supercritical CO_2_ conditions due to the increased CO_2_ solubility. Electrocarboxylation of 2-amino-5-bromopyridine and CO_2_ was also conducted in IL ([Bmim]BF_4_) in an undivided cell to give 6-aminonicotinic acid with 75% yield and 100% selectivity [108].

### Electrosynthesis of methylanilines

**Figure 9.**
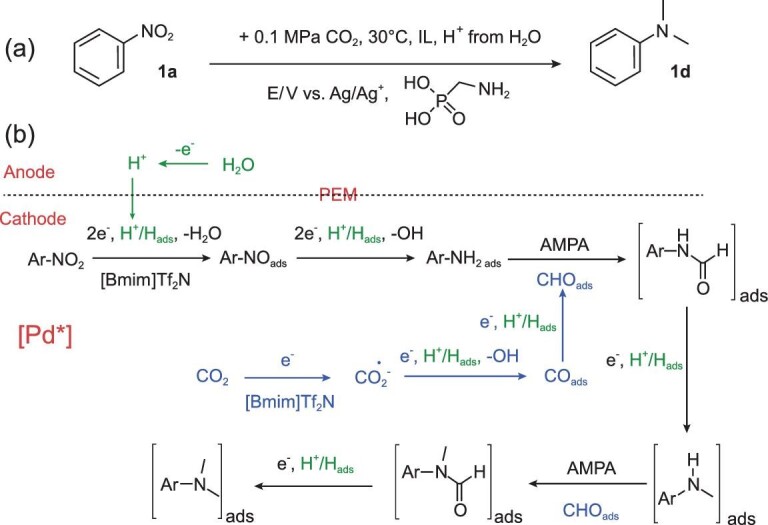
(a) Electrocatalytic methylation of nitrobenzene with CO_2_ and water over Pd_2.2_/Co–N/carbon catalysts and (b) the possible pathway. Adapted with permission from [109].

*N*-methylation reaction is very important in the chemical industry. Various valuable products, including dyes, pesticides and perfumes can be obtained by using methylanilines as intermediates. H_2_ or PhSiH_3_ is generally used as the reducing agent for the *N*-methylation reaction of anilines under high temperature and pressure. Recently, Sun *et al.* developed an electrochemical strategy for the synthesis of *N,N*-dimethylanilines from nitrobenzene and its derivatives, CO_2_, and water under ambient conditions (Fig. 9) [109]. H^+^ could be produced from water and act as a hydrogen source. Pd nanoparticles supported on Co-N/carbon were designed as the electrocatalysts, and 1-amino-methylphosphonic acid was used as the co-catalyst. The *N,N*-dimethylanilines were synthesized from the methylation of nitrobenzene and its derivatives in electrolyte composed of [Bmim]NTf_2_ and MeCN, and the corresponding products were obtained with high total yields of 71%–92%. By combining an electrocatalyst and a thermal catalyst, high yields of the desired products could be achieved with CO_2_ and water as the reactants under ambient reaction conditions making the scalable CO_2_ electroreduction coupled to organic synthesis possible.

## CONCLUSION AND PERSPECTIVE

Electrochemical conversion of CO_2_ into high-value carbonaceous chemicals and fuels by CO_2_ER and CO_2_EOT provides a promising strategy for achieving CO_2_ mitigation and relieving the dependence of our society on fossil fuels. With the high CO_2_ solubility and good electrolyte properties, ILs have been extensively explored for CO_2_ electrochemical conversion. Various types of ILs and IL-based mixtures have been studied, and imidazolium-based ILs are the most widely studied and used. The designability of ILs allows for the integration of functional ILs for CO_2_ conversion to achieve an optimum transformation pathway.

**CO_2_ER:** Significant research progress has been achieved in IL-based CO_2_ER systems. A diversity of electrocatalysts have been exploited and have achieved excellent catalytic performance. Many studies showed that lower overpotential, and higher current density and FE have been achieved in the presence of ILs. Experimental and theoretical studies have been conducted to figure out the reasons for the enhanced CO_2_ER efficiency by ILs. It is suggested that the interactions of IL with CO_2_ and reaction intermediates at the electrocatalyst surface contribute to the reduced activation energy and overpotential of CO_2_ER. The cations and anions of ILs have been screened to optimize the CO_2_ER efficiency. Imidazolium and pyrrolidinium cations have been proven to be very effective for enhancing CO_2_ER kinetics. The catalytic performance is influenced by complex interactions at the electric double layer, rather than simply by the chain length of the imidazolium cation and CO_2_ solubility. The cations of ILs play a multifunctional role in the electroreduction system, presumably acting as a co-catalyst, interacting with reaction intermediates, or changing the character of the interfacial double layer.

Despite the considerable progress achieved, challenges still exist in IL-based CO_2_ER systems. The kinetically sluggish multiple-electron transfer process attributes to the large overpotential and low current density. The product selectivity and yield, especially for value-added C2+ products, are still unsatisfactory for practical application. In addition, the actual role of ILs remains unclear. Optimized standard experimental systems and accurate fundamental theory are expected to be built in the future. Therefore, the development of more advanced IL-based systems is needed. The designability of ILs allows for optimizing electrocatalytic systems by adjusting various combinations of IL ions. The interplay between IL electrolyte and electrocatalysts can be engineered to facilitate CO_2_ER. Furthermore, more research efforts devoted to reaction kinetics are required to clarify the characteristics and underlying mechanism of IL-based electrocatalytic systems, especially the interactions at electrocatalyst–IL electrolyte interface. Also, the common reaction at the anode compartment of CO_2_ER is the OER; however, the OER as the anode reaction usually suffers from a large overpotential and generates a product with negligible economic value. Most recently, some studies have begun to explore alternative anode reactions for OER to lower the energy requirements for CO_2_ER and yield a higher-value anode product; this may provide a quite beneficial approach for improving the economics of CO_2_ER.

**CO_2_EOT:** CO_2_EOT in IL-based media could partially replace toxic reagents (e.g. CO and phosgene) and provide new routes to synthesizing a number of valuable chemicals, and the reaction was commonly conducted under mild reaction conditions. Therefore, it was considered as a green electrosynthesis methodology. We have reviewed recent advances in this area involving the reaction of CO_2_ with different substrates, like epoxides, alcohols, amines, aryl halides and olefins with the participation of ILs. ILs have a stabilization effect on the electro-induced CO_2_ molecule or substrates radical/anion intermediates, providing better control for succeeding reaction pathways and desirable products. The corresponding products of organic carbonates, carbamates and carboxylic acids are produced through electrosynthesis using CO_2_ and the substrates. As important chemicals in industry, organic carbonates (e.g. DMC) have received extensive research attention among these products. Various experimental parameters have been explored in order to achieve better catalytic performance, involving the IL components, electrocatalysts, electrochemical cell configurations and ion exchange membranes. Particularly, the electrosynthesis of DMC in IL-based media without additives has been carried out to avoid the use of toxic additives and simplify the separation system.

Although significant advances have been achieved in recent years, many challenges remain to be overcome. Firstly, the yield and selectivity of the products need to be improved for industrial application. The design of novel functional ILs and IL-based multi-component electrolytes could enhance CO_2_ conversion. Secondly, detailed information about the reaction mechanisms and the role of ILs should be elucidated to improve the catalytic activity and conversion efficiency. Thirdly, the CO_2_ conversion commonly studied in electrocatalytic systems is based on the formation of C−C, C−N and C−O bonds, which limits the development of this research area. Therefore, great research efforts should be devoted to the use of CO_2_ as C1 synthon to prepare more diverse chemicals, especially functional organic materials by the construction of different kinds of C−X bonds, like C−Si, C−P and C−S bonds. Much research work needs to be done to develop new reactions that are thermodynamically unfavorable by thermal catalysis, which is a very promising strategy for CO_2_ utilization.

In conclusion, electrochemical conversion of CO_2_ into value-added fuels and chemicals is a promising and rapidly developing area. Many studies have shown that ILs offer great potential for CO_2_ conversion technology. However, several critical challenges remain in this research area. Design of highly efficient catalyst-electrolyte-reactor systems, in-depth understanding of reaction mechanisms and the cooperative or synergistic effects of IL-based electrolytes and catalysts are crucial to tackling these challenges. Such research advances will promote the progress of industrialization of CO_2_ utilization.
